# Ketoprofen Associated with Hyaluronic Acid Hydrogel for Temporomandibular Disorder Treatment: An In Vitro Study

**DOI:** 10.3390/gels10120811

**Published:** 2024-12-10

**Authors:** Diego Garcia Miranda, Lucas de Paula Ramos, Nicole Fernanda dos Santos Lopes, Nicole Van Der Heijde Fernandes Silva, Cristina Pacheco Soares, Flavia Pires Rodrigues, Vinicius de Paula Morais, Thalita Sani-Taiariol, Mauricio Ribeiro Baldan, Luana Marotta Reis de Vasconcellos, Alexandre Luiz Souto Borges, Brigitte Grosgogeat, Kerstin Gritsch

**Affiliations:** 1 Multimaterials and Interfaces Laboratory (LMI), CNRS UMR 5615, University Claude Bernard Lyon 1, University of Lyon, 6 rue Victor Grignard, 69622 Villeurbanne, France; brigitte.grosgogeat@univ-lyon1.fr; 2 Department of Biosciences and Oral Diagnosis, Institute of Science and Technology, São Paulo State University, Avenida Francisco José Longo 777, São José dos Campos 12245-000, SP, Brazil; nf.lopes@unesp.br (N.F.d.S.L.); nicoleheijde30@gmail.com (N.V.D.H.F.S.); luana.marotta@unesp.br (L.M.R.d.V.); alexandre.borges@unesp.br (A.L.S.B.); 3 Laboratory “Health Systemic Process” (P2S), UR4129, Faculty of Medicine Laennec, University Claude Bernard Lyon 1, University of Lyon, 7 rue Guillaume Paradin, 69008 Lyon, France; lucas.de-paula-ramos@univ-lyon1.fr; 4 School of Dentistry, Federal University of Alfenas—UNIFAL. R. Gabriel Monteiro da Silva, 700—Centro, Alfenas 37130-001, MG, Brazil; 5 Laboratory of Cell Compartement Dynamics, Research and Development Institute, Paraíba Valley University, Avenida Shishima Hifumi 2911, São José dos Campos 12244-010, SP, Brazil; cpsoares@univap.br; 6 Oral Biology Division, School of Dentistry, Faculty of Medicine and Health, University of Leeds, Leeds LS2 9LU, UK; f.piresrodrigues@leeds.ac.uk; 7 Anton-Paar, Rua José de Magalhães 646, São Paulo 04026-090, SP, Brazil; vinicius.moraes@anton-paar.com; 8 National Space Research Institute, Avenida dos astronautas 1758, São José dos Campos 12227-010, SP, Brazil; thalita.taiariol@inpe.br (T.S.-T.); mauricio.baldan@inpe.br (M.R.B.); 9 Dental School, University Claude Bernard Lyon 1, University of Lyon, 7 rue Guillaume Paradin, 69372 Lyon, France; 10 Service d’Odontologie, Hospices Civils de Lyon, 8 Rue de l’Université, 69007 Lyon, France

**Keywords:** non-steroidal anti-inflammatory agents 1, polysaccharides 2, osteoarthritis 3

## Abstract

Temporomandibular disorders (TMD) are a public health problem that affects around 12% of the global population. The treatment is based on analgesics, non-steroidal anti-inflammatory, corticosteroids, anticonvulsants, or arthrocentesis associated with hyaluronic acid-based viscosupplementation. However, the use of hyaluronic acid alone in viscosupplementation does not seem to be enough to regulate the intra-articular inflammatory process. So, we propose to develop and evaluate the physicochemical and biological properties in vitro of hyaluronic acid hydrogels (HA) associated with ketoprofen (KET) as a new therapeutic treatment for TMD. The hydrogels were synthesized with 3% HA and 0.125, 0.250, 0.500, or 1% KET. Physicochemical analyses of Attenuated Total reflectance-Fourier transform infrared spectroscopy (FTIR), Thermogravimetry (TGA), Rheology by Frequency, Amplitude sweeps, temperature ramp, and scanning electron microscopy (SEM) were performed with or without sterilization and cycled. Cytocompatibility and genotoxicity (micronucleus assay) were performed in mouse macrophages (RAW 264-7) for 24 h. Results: FTIR spectrum showed characteristic absorptions of HA and KET. In the TGA, two mass loss peaks were observed, the first representing the water evaporation at 30 and 100 °C, and the second peaks between 200 and 300 °C, indicating the degradation of HA and KET. Rheology tests in the oscillatory regime classified the hydrogels as non-Newtonian fluids, time-dependent, and thixotropic. Mouse macrophages (RAW 264-7) presented viability of 83.6% for HA, 50.7% for KET, and 92.4%, 66.1%, 65.3%, and 87.7% for hydrogels, in addition to the absence of genotoxicity. Conclusions: Hyaluronic acid associated with ketoprofen shows satisfactory physicochemical and biological properties for use as viscosupplementation. As a limiting point of this study, further research is needed to evaluate the pharmacodynamic, toxicological, and pharmacokinetic characteristics of a complete organism

## 1. Introduction

The American Academy of Head, Neck and Facial Pain defines temporomandibular disorders (TMD) as a comprehensive term, being a set of pathophysiological conditions that affect the musculoskeletal and neuromuscular structures of the masticatory muscles and temporomandibular joint (TMJ) [1]. TMD is an important public health problem with a variant distribution, affecting around 6 to 12% of the global population, and is more frequent in women between the ages of 20 and 40 [2]. TMD is also considered the most common cause of chronic pain of non-dental origin in the orofacial area [3]. 

Osteoarthritis of the temporomandibular joint is one of the most common forms of temporomandibular dysfunction and can be described as a low-inflammation arthritic condition when compared to systemic arthritis [4]. The pathophysiological process of TMD is complex because although it is defined as a local inflammatory disease, its development can be due to local and systemic factors [5]. Local factors include microtrauma secondary to bruxism, parafunctional habits, and improper displacement of the articular disc. Systemic factors include rheumatoid, psoriatic, and reactive arthritis [6,7]. This highlights the importance of the host’s immune-inflammatory response in the osteoarthritic process [8]. 

Regarding patients’ pain, Sebastiani et al. [9] showed that the genetic polymorphism of IL-6 in some patients leads to an increase in the expression of this cytokine, contributing negatively to the sensitivity and duration of pain. The Consortium for Research on Genetics and Quality of Life suggested that the IL-6 production gene is associated with overall quality of life, where the interaction of the cytokine with its receptors generates inflammation, activation of the vagal afferent nerve, and promotes the severity and duration of pain, as well as triggering depression [10]. 

The condition has an established impact on social, economic, and health structures. Studies point to reduced levels of health-related quality of life, socialization, and social networks, together with increased depression among TMD patients [11,12,13]. Pain and dysphagia affect both the physical functioning and emotional well-being of the patient; this manifests as a reduction in the ability to consume typical diets and decreased participation in social events [14].

TMD treatment is based on the patient’s clinical manifestations. Today, because it is understood that the immunoinflammatory process plays a fundamental role in the pathogenesis of the disease, interventions are based on the use of analgesics, non-steroidal anti-inflammatory drugs, corticosteroids, muscle relaxants, benzodiazepines, antidepressants, and anticonvulsants [15,16]. 

Arthrocentesis, a minimally invasive surgical procedure, aims to reduce the concentration of pro-inflammatory cytokines in the synovial fluid and cleave possible adhesions in the articular disc [17,18,19]. Arthrocentesis can also be associated with hyaluronic acid-based viscosupplementation to restore the biomechanical properties of the synovial fluid [20,21,22,23,24]. Viscosupplementation is a therapy that consists of an intra-articular injection of sodium hyaluronate (HA) into the joint compartments, applied to promote improvement in mandibular mobility, joint homeostasis, and friction decay, stimulating type B synoviocytes to produce additional synovial fluid, which results in improvements in the rheological properties of the synovial fluid, superior lubrication, absorption of functional loads and stimulation of chondrocyte proliferation in fibrocartilage (januzzi). However, the use of hyaluronic acid (HA) alone does not seem to be enough to regulate the intra-articular inflammatory process [16,25].

As for the principles of hyaluronic acid, it is a natural polysaccharide present in various tissues of the human body, including the skin, cartilage, and synovial fluid [26]. It consists of a linear molecule made up of repeated disaccharide units composed of D-glucuronic acid and N-acetylglucosamine [27,28]. It has several biological functions, including regulating tissue hydration and lubrication, maintaining the structural integrity of tissues, and interacting with cells via specific receptors such as CD44 [29,30]. Hyaluronic acid is also an important component in the formulation of hydrogels since its ability to form bonds allows the crosslinking of polymers, resulting in materials with adjustable mechanical and drug-release properties [31,32,33].

Ketoprofen (KET) is a propionic acid derivative non-steroidal anti-inflammatory drug (NSAID) with analgesic and antipyretic properties [34,35]. In various branches of medicine and dentistry, KET is used to reduce pain associated with inflammatory processes and to treat chronic pain [36]. The indole methyl moiety in KET is involved in the enzyme-inhibiting properties. KET selectively inhibits cyclooxygenase 1 and 2 and blocks prostaglandin synthesis at the peripheral level [37]. Also, in recent studies, KET has been shown to be the therapy of choice for treating osteoarthritis [38,39]. We hypothesize that the combination of the biomechanical properties of the hyaluronic acid hydrogel and the immunomodulatory effect of KET could improve the success of treatment by temporomandibular dysfunction. Therefore, the aim of this study was to evaluate the physicochemical and biological properties in vitro of HA hydrogels associated with ketoprofen to propose a new therapeutic compound.

## 2. Results and Discussion

### 2.1. Attenuated Total Reflectance-Fourier Transform Infrared Spectroscopy (FTIR)

The FTIR spectrum of HA showed characteristic absorptions at 3300 cm^−1^ stretching of (OH) and (NH), 2882 cm^−1^ stretching (C-H) presented in lines 1 and 2; 1600 cm^−1^ asymmetric stretching of carboxyl groups (C = O) presented in line 4; 1560 cm^−1^ (amine II); 1402 cm^−1^ symmetric stretching of carboxyl groups (CO); 1148 cm^−1^, the C-O-C group (O bridge); 1080 cm^−1^ exocyclic groups (C-O) and (C-C); and 1036 cm^−1^ (C-OH) [40,41,42,43].

The FTIR spectrum of KET showed characteristic absorptions at 1701 cm^−1^ acid carbonyl group (C=O), 1660 cm^−1^ ketone carbonyl group (C=O) [44], 1600 cm^−1^ aromatic stretching (C=C) presented in line 4, 1431 cm^−1^ CH-CH_3_ deformation, 1080 cm^−1^ exocyclic groups (C-O) and (C-C), 1036 cm^−1^ (C-OH), 716 and 966 cm^−1^ out-of-plane deformation (C-H) [45,46,47,48]. 

**Figure 1:**
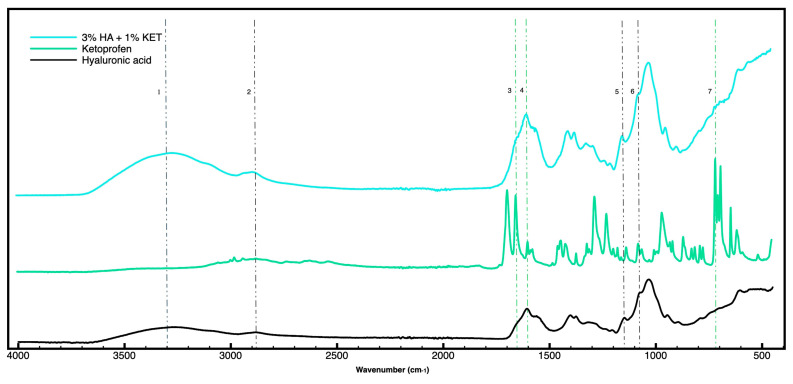
FTIR spectra of HA (line black), KET (line green), and hydrogel 3% HA associated with 1% KET (line blue). The vertically dotted black lines indicate the molecular signatures of HA present in the isolated molecule and in the gel combined with KET. The vertically dotted lines in green indicate the molecular signatures of KET present in the isolated molecule and in the gel combined with HA.

The FTIR spectrum of the gel (3% HA + 1% KET) showed characteristic absorptions at 3300 cm^−1^ stretching of (OH) and (NH); 2882 cm^−1^ stretching (C-H) presented in lines 1 and 2 in Figure 1; 1660 cm^−1^ ketone carbonyl group (C=O); 1600 cm^−1^ aromatic stretching (C=C) or asymmetric stretching of carboxyl groups (C = O) presented in lines 3 and 4; 1148 cm^−1^, the C-O-C group (O bridge); and 1080 cm^−1^ exocyclic groups (C-O), shown in lines 5 and 6; absorption in 716 cm^−1^ representative of out-of-plane deformation (C-H), shown in line 7. These results suggest the presence of both compounds in the hydrogel sample 3% HA + 1% KET without a spontaneous reaction between the compounds.

### 2.2. Thermogravimetric Analysis (TGA)

**Figure 2:**
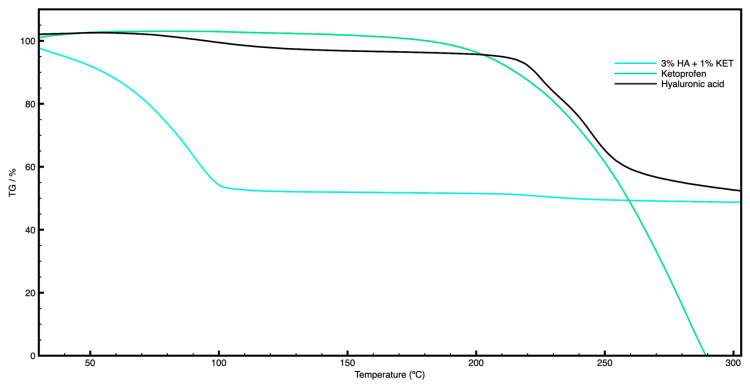
Analysis of the decomposition of masses in relation to temperature. TGA spectra of HA (line black), KET (line green), and hydrogel 3% HA associated with 1% KET (line blue).

The thermogravimetric analysis graph of hyaluronic acid shows one peak of mass drop between 220 and 260 °C, representing 40% of the total mass [49]. The thermogravimetric analysis graph of ketoprofen shows one peak of mass drop between 200 and 290 °C, representing 100% of the mass [50]. The thermogravimetric analysis graph of 3% HA + 1% KET shows two regions of mass drop, the first between 30 and 100 °C, representing the evaporation of the water mass of the hydrogel, and the second between 200 and 300 °C, representing the degradation of hyaluronic acid and ketoprofen, expressed in Figure 2.

It is known that the hyaluronic acid molecule at neutral pH attracts water molecules up to 10,000 times its mass. The water molecules interact with the hyaluronic acid through hydrogen bonds, which stabilize the molecular structure of the hydrogel. When the hydrogel is heated, the kinetic energy and, consequently, the thermal energy of the solution increases, leading to greater agitation of the molecules, breaking the hydrogen bonds, thus allowing the evaporation of the water. 

Given that the human body temperature is around 36 to 37.5 °C, it is expected that the hydrogel will lose mass due to water loss. However, the hydrogel would be in a closed system (temporomandibular joint), which would reduce the dispersion of water molecules into the environment. In addition, the human body has physiological mechanisms for maintaining tissue hydration, which would contribute to maintaining the hydration of the hydrogel. Although hyaluronic acid is hydroscopic, the hydrogel will have a volumetric limitation determined by the size of the joint capsule, thus limiting water absorption.

Regarding hyaluronic acid molecules and ketoprofen, there is no impact in the intracorporeal environment, given their high thermodegradation temperatures.

### 2.3. Rheology Assay

#### 2.3.1. Frequency Sweep

**Figure 3:**
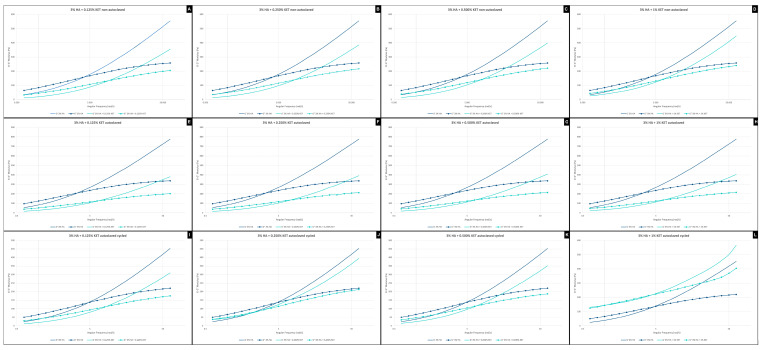
Frequency sweep of 3% HA + 0.125% KET hydrogel non-autoclaved (**A**), 3% hyaluronic acid HA + 0.250% KET hydrogel non-autoclaved (**B**), 3% HA + 0.500% KET hydrogel non-autoclaved (**C**), 3% HA + 1% KET hydrogel non-autoclaved (**D**), 3% HA + 0.125% KET hydrogel autoclaved (**E**), 3% HA + 0.250% KET hydrogel autoclaved (**F**), 3% HA + 0.500% KET hydrogel autoclaved (**G**), 3% HA + 1% KET hydrogel autoclaved (**H**), 3% HA + 0.125% KET hydrogel autoclaved cycled (**I**), 3% HA + 0.250% KET hydrogel autoclaved cycled (**J**), 3% HA + 0.500% KET hydrogel autoclaved cycled (**K**), 3% HA + 1% KET hydrogel autoclaved cycled (**L**).

The frequency analyses of non-autoclaved hydrogels (Figure 3A–D) indicate a higher elastic modulus for the hyaluronic acid alone sample (3% HA), while the addition of ketoprofen reduces the viscoelasticity (G′)—a similar effect was observed in the study by Kurbasic et al. [51]. When we evaluated only the hydrogels associated with ketoprofen, it was possible to see that the viscoelastic modulus gradually increased with the concentration of the anti-inflammatory added. It was also possible to see that the addition of ketoprofen increases the gel’s stability over time. 

The autoclaving process causes the hydrogels to stiffen, a phenomenon observed in the initial values of G′ and G″, making the material more viscoelastic. The comparison of stability between autoclaved and non-autoclaved materials shows no differences. 

The cycling process applied to the autoclaved hyaluronic acid gel (3% HA) showed a reduction in the viscoelasticity modulus with G′ starting at 450 Pa, compared to 800 Pa for the autoclaved material alone and 550 Pa for the synthesized material alone. It can also be seen that the 3% HA + 1% KET sample (Figure 3L) maintains the ratio between G′ and G″ but shows a gain in viscoelasticity, with an increase in the G′ modulus, giving viscoelasticity and stability superior to the 3%HA group.

Fam et al., 2007 [52] evaluated the viscosity of knee synovial fluids, finding values from 1 to 175 Pa for healthy patients and 0.1 to 1 Pa for patients with diagnosed osteoarthritis. In the present work, it is possible to verify that the hydrogel associated with ketoprofen presents viscosity characteristics at zero shear rates of 150 Pa for the hydrogels containing 3% AH + 0.125% KET and 3% AH + 0.250 KET, and 170 Pa for the hydrogels 3% AH + 0.500% KET and 3% AH + 1% KET. These values show a similarity to the consistency of human joint fluids. It is important to highlight that studies that evaluate the viscosity of the synovial fluid of the temporomandibular joint are scarce in the literature, with no rheological data being found for discussion.

#### 2.3.2. Amplitude Sweep

The non-autoclaved isolated hyaluronic acid gel (3%HA) has a higher stiffness than the samples associated with ketoprofen, as shown by the distance between the G′ and G″ moduli or even by the state of flow, in which the gel shows deformation near the 2% region. This result goes against that found in the study by Otto & Froelich [53], who observed no change in the amplitude curve after adding ketoprofen. This dissimilarity can be explained by the difference in the base polymer used to form the hydrogel, which, in our study, was HA, and in Otto & Froelich’s study, was Carbopol ^®^.

**Figure 4:**
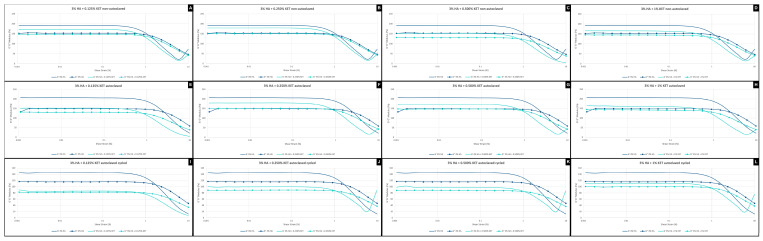
Amplitude sweep of 3% HA + 0.125% KET hydrogel non-autoclaved (**A**), 3% HA + 0.250% KET hydrogel non-autoclaved (**B**), 3% HA + 0.500% KET hydrogel non-autoclaved (**C**), 3% HA + 1% KET hydrogel non-autoclaved (**D**), 3% HA + 0.125% KET hydrogel autoclaved (**E**), 3% HA + 0.250% KET hydrogel autoclaved (**F**), 3% HA + 0.500% KET hydrogel autoclaved (**G**), 3% HA + 1% KET hydrogel autoclaved (**H**), 3% HA + 0.125% KET hydrogel autoclaved cycled (**I**), 3% HA + 0.250% KET hydrogel autoclaved cycled (**J**), 3% HA + 0.500% KET hydrogel autoclaved cycled (**K**), 3% HA + 1% KET hydrogel autoclaved cycled (**L**).

The samples associated with KET show a decrease in elastic modulus. As an example, the 3% HA + 0.250% KET sample (Figure 4B) has a linear viscoelastic region, with a deformation of 1% to enter the flow state when G″ is higher than G′, i.e., fluidized. Among all the gel concentrations, the 3% HA + 0.500% KET (Figure 4C) sample shows the greatest reduction in viscosity, with G′ and G” modules very close to each other, almost becoming a viscoelastic liquid.

The autoclaving process did not cause any significant changes in the amplitude analysis of the gels associated with ketoprofen. The only point to note is that the sample isolated with HA (3% HA) has a G′ modulus of more than 200 Pa, while the non-autoclaved sample has a G′ modulus of less than 200 Pa, which indicates an increase in the modulus of elasticity. 

When observing all the groups (Figure 4E–H) after the aging process (cycling), it is possible to see that the gels suffer a decrease in the modulus of elasticity (G′), showing polymer relaxation. The values measured in the G′ modulus start at around 150 Pa, while the sample autoclaved without undergoing the cycling process has a G′ modulus of over 200 Pa. 

It was also possible to see that the addition of KET to the HA gel reduces the modulus of elasticity, where G′ is around 150 Pa, while the 3% HA + 1% KET sample has a G′ of around 110 Pa. The decrease in modulus of elasticity is proportional to the decrease in KET concentration, in which the 3% HA + 0.125% KET (Figure 4E) group shows G′ in the order of 90 Pa.

The amplitude analyses indicate that the stress process promoted by cycling (Figure 4I–L) has an impact on the elastic characteristics of the hydrogels, making them more fluid.

#### 2.3.3. Temperature Ramp

**Figure 5:**
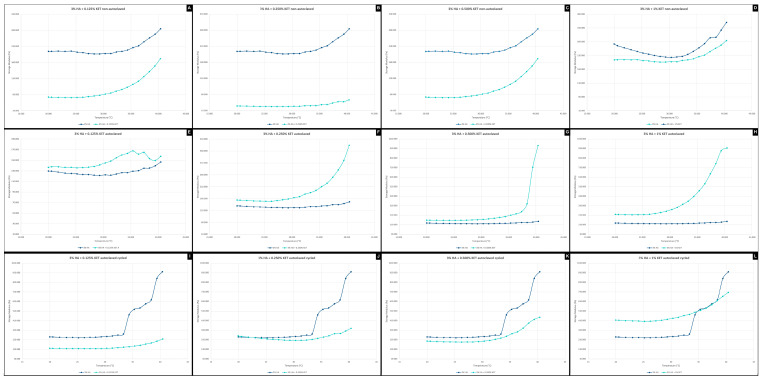
Temperature ramp of 3% HA + 0.125% KET hydrogel non-autoclaved (**A**), 3% HA + 0.250% KET hydrogel non-autoclaved (**B**), 3% HA + 0.500% KET hydrogel non-autoclaved (**C**), 3% HA + 1% KET hydrogel non-autoclaved (**D**), 3% HA + 0.125% KET hydrogel autoclaved (**E**), 3% HA + 0.250% KET hydrogel autoclaved (**F**), 3% HA + 0.500% KET hydrogel autoclaved (**G**), 3% HA + 1% KET hydrogel autoclaved (**H**), 3% HA + 0.125% KET hydrogel autoclaved cycled (**I**), 3% HA + 0.250% KET hydrogel autoclaved cycled (**J**), 3% HA + 0.500% KET hydrogel autoclaved cycled (**K**), 3% HA + 1% KET hydrogel autoclaved cycled (**L**).

The temperature ramp of the non-autoclaved gels indicates an intermediate elastic modulus characteristic at 210 Pa. The concentrations of 3% HA + 0.125% KET (Figure 5A) and 3% HA + 0.250% KET (Figure 5B) show low viscosity, while the concentrations of 3% HA + 0.500% KET (Figure 5C) and 3% HA + 1% KET (Figure 5D) show higher viscosity than the sample of hyaluronic acid alone (3% HA). 

The process of autoclaving the samples suggests a molecular rearrangement of the polymer, in which the gradual increase in the concentration of KET affects the increase in the viscosity profile. This can be seen in Figure 5, with emphasis on the 1% concentration (Figure 5E), which shows an upward viscosity ramp from a temperature of 25°C. 

The groups that were autoclaved with subsequent cycling (Figure 5I–L) show the phenomenon of material aging, with a reduction in the rigidity and elastic memory of the material, when compared to the materials that were autoclaved only. García-Villegas et al. also observed the process that affected the viscosity values the most was temperature cycling due to the abrupt temperature changes [54]. The 3% HA + 1% KET (Figure 5I) sample shows stabilization in the elastic modulus, with no increase in stiffness as the temperature rises, i.e., an attenuation of the elastic modulus as the temperature varies.

### 2.4. Scanning Electron Microscopy with Field Emission Gun (SEM-FEG)

**Figure 6:**
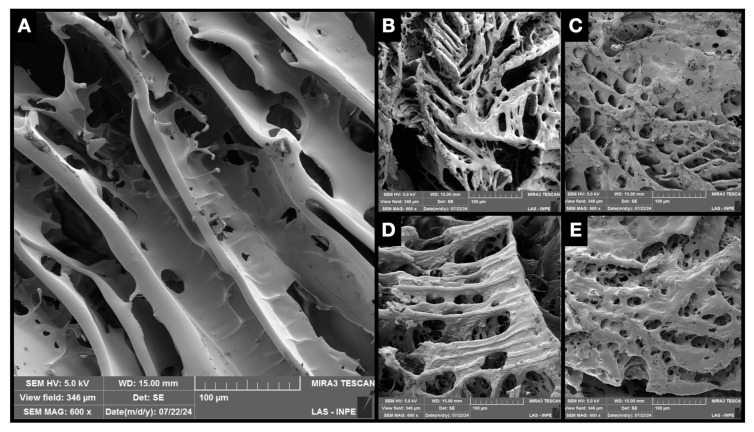
Micrography of 3% HA hydrogel (**A**), 3% HA + 0.125% KET hydrogel (**B**), 3% HA + 0.250% KET hydrogel (**C**), 3% HA + 0.500% KET hydrogel (**D**), 3% HA + 1% KET hydrogel (**E**).

In scanning electron microscopy with a field emission gun, no structural difference could be observed between the isolated hyaluronic acid hydrogels when compared to those associated with ketoprofen (Figure 6). SEM micrographs revealed a sheet-like structure containing pores of various sizes and interconnected with a three-dimensional network, with spacing between the hydrogel layers. The micrograph in Figure 6E shows a structure composed of smaller pores and fewer spaces between the layers. Structures shown in Figure 6B–E have a rougher appearance than the ones shown in Figure 6A. The porosity of the gels was not evaluated metrically since the drying process influences the crosslinks and, therefore, the porosity of the material [55].

### 2.5. Cell Viability by Resazurin Essay

**Figure 7:**
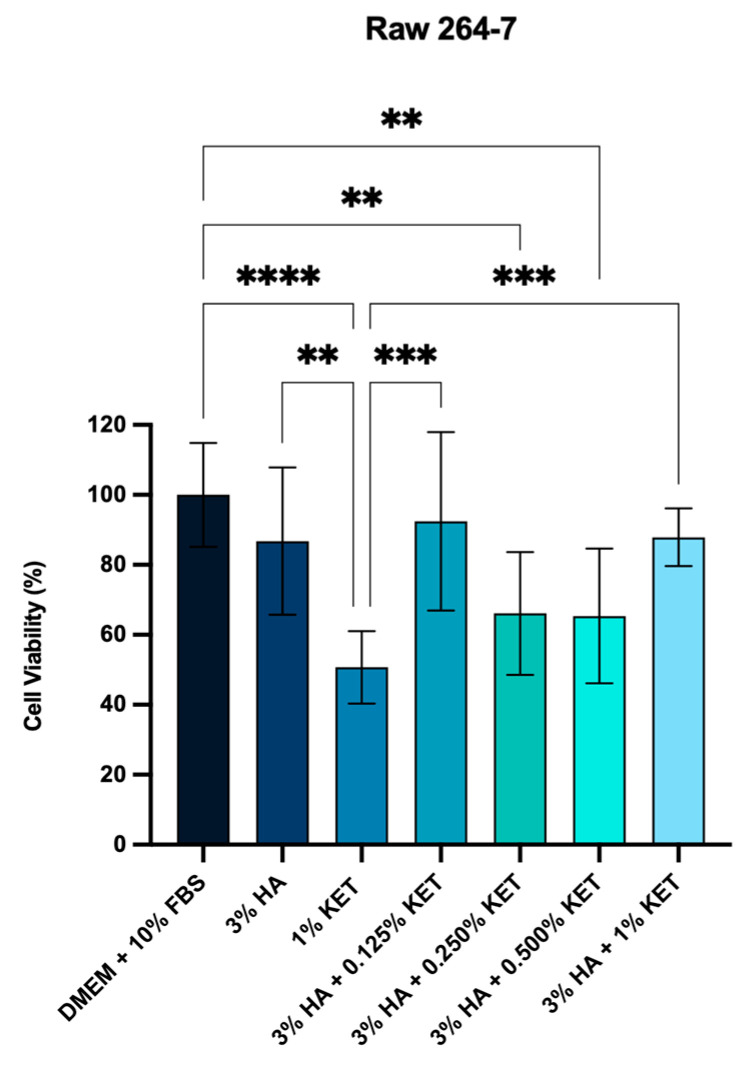
Cytocompatibility by hydrogels on mouse macrophages (RAW 264.7). *p* < 0.0021 (**), *p* < 0.0002 (***), *p* < 0.0001 (****).

After contact for 24 h with the 3% HA hydrogel, mouse macrophages (RAW 264.7) presented cell viability of 83.6%, while the HA hydrogels conjugated to concentrations of 0.125, 0.250, 0.500, and 1% KET presented viability of 92.4%, 66.1%, 65.3%, and 87,7%. The application of 1% ketoprofen resulted in 50.7% cell viability, as shown in Figure 7.

Recent studies have sought to improve the availability, solubility, and compatibility of KET as an anti-inflammatory agent associated with materials. Chachlioutaki et al. evaluated the cytotoxic activity of zinc oxide (ZnO) nanofibers associated with KET at concentrations of 0.5 to 1 mg/mL. The polymer based on 3% hydroxypropylmethyl cellulose (HPMC) associated with 1 mg/mL of KET exhibited variations of 30 to 40% viability with the times of 24, 48, and 72 h of application on mouse macrophages (RAW 264-7). In contrast, the present study shows that the HA polymer associated with KET presents greater cytocompatibility with the macrophage lineage (RAW 264.7), exhibiting viability greater than 60%.

Criado-Gonzalez [56] evaluated the application of hybrid hydrogels based on phosphorylated tripeptides Fmoc-FFpY (F: phenylalanine, pY: phosphorylated tyrosine) associated with KET. The gel at a concentration of 0.5%, applied by direct contact, showed cell viability higher than 85% on mouse macrophage (RAW 264.7) and human dermal fibroblast (HDF) lines. The results corroborate the present study, indicating cell viability ranging from 83.6% to 60.3% on the macrophage line. It was possible to verify that concentrations of 0.125 and 1.0 demonstrate the best viability values, indicating the absence of toxicity to the mouse macrophage lineage.

Moses et al. [57] evaluated the application of KET in the synovial membranes of horses after stress by Lipolysaccharides (LPS). The tissues were cultured in the laboratory under aseptic conditions and incubated with 0.001 μg of LPS/mL for tissue inflammation. The evaluation demonstrated that the administration of 4 mg/mL of KET promoted a reduction of 1 pg/mL in prostaglandin production, in addition to not promoting significant changes in the production of hyaluronic acid and maintaining preserved tissue characteristics. Wyns et al. [57] reinforce the low cellular toxicity, as well as the anti-inflammatory activity of KET, showing the reduction in prostaglandins in pig monocytes, besides indicating the decrease in Interleukin 6. The findings of Moses et al. [57] and Wyns et al. [58] corroborate the present study, with a view to the clinical application of HA gels associated with KET, supporting cytocompatibility in cartilage tissue cells and mouse macrophages, additionally to highlighting the anti-inflammatory activity promoted by the non-steroidal drug.

### 2.6. Genotoxicity Assay by Micronucleus

The genotoxicity evaluation for the DMEM + 10% BFS, 3% HA, and 3% HA + 0.250% KET groups showed the formation of two micronuclei. The 1.0% KET, 3% HA + 0.500% KET and 3% HA + 0.125% KET groups showed three micronuclei. The 3% HA + 1.0% KET group showed the formation of five micronuclei, while the positive genotoxicity control caused the formation of 29 micronuclei.

The evaluation of the genotoxicity of KET and HA by micronuclei is scarce in the literature, making this work pioneering in this regard. Philipose et al. [59] evaluated the mutagenic potential of KET using the AMES test (in vitro) and the evaluation of sister chromatid exchange (SCE) in cells extracted from the bone marrow of rats (in vivo). The results of the AMES test, using the Salmonella strains TA97a, TA100, and TA102, demonstrated the toxicity of the drug at a concentration of 5000 µg, at a threshold of concentrations of 1, 10, 100, 1000, and 5000 µg. The authors indicate the inconclusiveness of this evaluation because the same result was not obtained when the test was repeated. 

The in vivo evaluation involved the administration of 270 mg/kg of KET to rat bone marrow cells, resulting in an average of 5.14 cells with chromatid exchange in a total of 150 cells evaluated, a value close to the control group (-), which presented 4.68 cells. In contrast, the group presented with the genotoxic drug (Cyclophosphamide 10 mg/kg) presented an average of 19.86 cells with chromatid exchange in a total of 150 cells evaluated. The authors conclude that ketoprofen has low genotoxic potential in in vivo tests [59]. These results corroborate the present study, which, despite using a different methodology, contributes to the literature, with results indicating the absence/low formation of micronuclei in mouse macrophages exposed to 1 mM/mL of KET.

**Figure 8:**
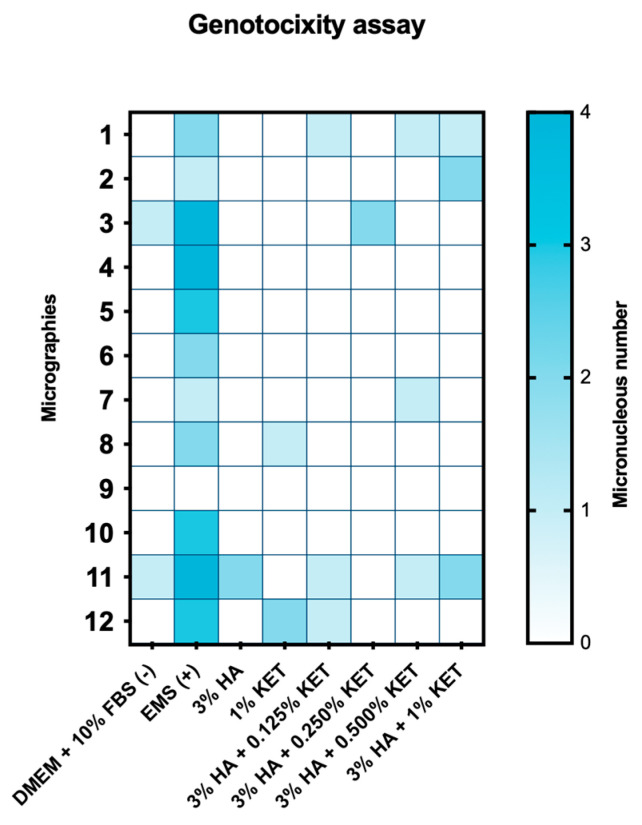
Genotocixity assay by micronucleus.

Salústio et al. [60] reinforce the absence of genotoxicity of KET in relation to human metabolism. The researchers evaluated the activity of reactive acyl glucuronides, a molecule derived from the metabolism of UDP-glucuronosyltransferases (UGTs) that performs the hepatic excretion of xenobiotics on KET. The results conclude that KET presents cellular toxicity, obtaining less than 50% of predictions of mouse hepatocytes with a concentration of 0.12 mM of KET. However, the comet test shows that the drug does not present genotoxicity, presenting extraction of genetic material similar to the control group. These results corroborate the present study, where cellular observation of mouse macrophages was observed in 50.4% after the application for 24 h of 1 mM of KET and the absence of genotoxicity after the application of the same parameters, observed in Figure 8.

As a limiting point of this study, it is worth noting that the biological analyses were performed on a single type of cell in a monolayer, exclusively evaluating the metabolic behavior of an immune cell (macrophages). Further research is needed to evaluate the application of the drug in chondrocytes and synoviocytes, exhausting the pharmacodynamic profile of the drug in vitro. In addition, it is necessary to use in vivo tests to evaluate the toxicological and pharmacokinetic characteristics of a complete organism. 

## 3. Conclusions

Hyaluronic HA associated with KET shows satisfactory physicochemical and biological properties for use as viscosupplementation. The 3% HA + 1.0% KET hydrogel seems to be the best choice since it has the highest concentration of KET and shows no significant biophysicochemical changes when compared to the controls. In vivo tests are necessary to determine the pharmacokinetics, pharmacodynamics, and lethal dose of the drug before clinical trials can begin to ascertain their therapeutic applicability.

## 4. Materials and Methods

### 4.1. Chemical Reagents

Hyaluronic acid (HA) (CAS nº: 9004-61-9, purity: 95.9%, molecular weight: 1.300.000 Dalton, Inlab Analitic^®^, code: HA2022042345X, São Paulo, Brazil), ketoprofen (CAS nº: 22071-15-4, purity: 99.08%, Lepuge^®^, code: KPO-2105014), phosphate buffer saline (PBS) (code: P2272, Sigma-Aldrich^®^, St. Louis, MO, USA), sodium hydroxide (NaOH) (CAS nº: 1320-73-2, code: S5881, purity: 98%, Sigma-Aldrich^®^, St. Louis, MO, USA), formic acid (CAS nº: 64-18-6, code: 27001, purity:98%, Sigma-Aldrich^®^, St. Louis, MO, USA), methanol (CAS nº: 67-56-1, purity: 99.8%, Synth^®^, Diadema, Brazil), Eagle’s medium modified by Dulbecco—DMEM (LGC Biotechnology^®^, Cotia, Brazil), Fetal Bovine Serum—FBS (Invitrogen^®^, New York, NY, USA), 7-Hidróxi-3H-fenoxazin-3-ona-10-óxido (Resazurin) (CAS nº: 62758-13-8, code: R1017, Sigma-Aldrich^®^, St. Louis, MO, USA), Ethyl Methane sulfonate (EMS) (CAS nº: 62-50-0, code: M08080, Sigma-Aldrich^®^, St. Louis, MO, USA), Cytochalasin B (CAS nº: 14930-96-2, code: C2743, purity: 98%, Sigma-Aldrich^®^, St. Louis, MO, USA), 4′,6-Diamidino-2-fenilindol, 2-(4-Amidinofenil)-6-indolecarbamidina with fluoroshield (DAPI) (CAS nº: 28718-90-3, code: F6057, Sigma-Aldrich^®^, St. Louis, MO, USA), Ethanol (CAS nº: 64-17-5, purity:99.5%, Synth^®^, Diadema, Brazil).

### 4.2. Equipments

Class II biological safety cabinet (Veco^®^, biosseg-06, Sumaré, São Paulo, Brazil), analytical balance (Mettler Toledo^®^, Balance XPR106DUH/A, Columbus, OH, USA), type I ultrapure water purification system (Allcron^®^, direct-Pure^®^ Genie, São Paulo, Brazil), autoclave (Cristofoli Biossegurança^®^, Vitale21, Campo Mourão, Parana, Brazil), dual asymmetric centrifuge mixer (Hauschild^®^, Speed MixerDAC150. 1 FVZ, Waterkamp, Hamm, Germany), Ph meter (Digimed^®^, DM-20, São Paulo, Brazil), Spectrometer Matrix Assisted Laser Desorption Ionization-Time of Flight—MALDI-TOF-MS Ettan (Amersham Biosciences^®^, Amersham, UK), high-performance liquid chromatography with a photodiode detector instrument (HPLC) (Merck-Hitachi^®^, D-7000, Tokyo, Japan), freezer for cryogenesis (Freezer -80) (NuAire Laboratory Equipement^®^, Glacier polar edition -86 ultra-low temperature freezer, Plymouth, MA, USA), freeze dryer (Terroni^®^, LS3000, São Carlos, São Paulo, Brazil), Fourier transform infrared spectroscopy (FTIR) (Perkin Elmer^®^, LR64912C, Waltham, MA, USA), thermogravimetric analyzer (TGA) (NETZSCH^®^, STA449F1 Jupiter, Selb, Bavaria, Germany), dynamic shear rheometer (Anton-paar^®^, SmartPave 92, Graz, Austria), Sputter Coater (Quorum Technologies^®^, Emitech—SC7620, Kent, UK); Scanning Electron Microscope with Field Emission Gun (FEG^®^, TESCAN MIRA 3^®^, Brno, Czech Republic), CO_2_ Incubator (Sanyo^®^, MCO-19AIC(UV), Osaka, Japan), water bath precision (TermoFisher Scientific^®^, TSGP02, Waltham, MA, USA), refrigerated centrifuge (Labnet^®^, HEREMLE Z300^®^, Madrid, Spain), inverted microscope (Ziess^®^, Axiovert 40C, Jena, Thuringia, Germany), fluorescence microscope (Ziess^®^, Axio Observer A1, Jena, Thuringia, Germany), spectrophotometer (Lonza Biotek^®^, ELX808LBS, Winooski, VT, USA), thermal cycler (Nova Ética^®^, 521-D, São Paulo, Brazil). 

### 4.3. Hydrogels Preparation

A 3% (*w*/*v*) solution of HA was prepared in ultra-pure type I water for physico-chemical analysis and biological analysis. Then 1%, 0.5%, 0.25% or 0.125% KET (*w*/*w*) was added. The hydrogel was homogenized in a mixer for 1 min and the pH was adjusted to 7.2 with NaOH.

### 4.4. Sterilization and Thermal Fatigue 

The gels were sterilized in an autoclave for 5 min at 118 °C [61]. 

Sterilized hydrogels were subjected to thermal fatigue cycles in a controlled simulator. Simultaneously, the specimens were thermocycled between 5 °C and 55 °C, maintained at each temperature for 30 s, totaling 1 min per cycle. A total of 1000 thermal cycles were performed in a total of 2 days stored in the device [62].

### 4.5. Freeze-Drying

The hydrogel samples were frozen at −80 degrees for 24 h and then placed in a freeze-dryer for 3 days to sublimate the solvent.

### 4.6. Attenuated Total Reflectance-Fourier Transform Infrared Spectroscopy (FTIR)

The freeze-dried samples were analyzed by FT-IR, obtaining wave number spectra between 500 and 4000 cm^−1^, with a nominal resolution of 4 cm^−1^ and 100 scans per sample.

### 4.7. Thermogravimetric Analysis (TGA)

The samples of the gels were analyzed by thermogravimetry, obtaining the thermal analysis curves by scanning the temperature range between 30 and 300 °C, with a rate change of 10 °C/min in an atmosphere of argon (Ar).

### 4.8. Rheology Assay

#### 4.8.1. Frequency Sweep

In the frequency ramp, after the sample had settled, we waited 2 min for the hydrogels to recover and relax. Shear deformation was carried out with a 1% constant and frequency decreasing from 10 to 0.1 hertz.

#### 4.8.2. Amplitude Sweep

In the amplitude ramp, after the sample had settled, we waited 2 min for the polymers to recover and relax. A controlled shear deformation ramp was applied from 0.001 to 100% with a frequency of 10 radians/second, with a fixed frequency.

#### 4.8.3. Temperature Ramp

The sample was preheated to 20 degrees Celsius for 2 min, after which the temperature ramp test was applied with a controlled deformation ramp and frequency, with a deformation of 1%, a frequency of 10 radians/second, and a heating rate of 1 degree Celsius per minute.

### 4.9. Scanning Electron Microscopy with Field Emission Gun (SEM-FEG)

For SEM, the lyophilized hydrogels were transferred to aluminum stubs and covered with gold for 120 s at 40 mA. After the process, the discs were analyzed and photographed by the scanning electron microscope. Then, the metallized SEM samples were used for surface elemental characterization by low energy dispersive X-ray cartography technique. 

### 4.10. Biological Performance on In Vitro Cell Cultures

Cytocompatibility was carried out on mouse macrophages (RAW 264-7). Cell culture was carried out in DMEM supplemented with 10% FBS, incubated in an oven at 37 °C, with atmospheric humidity and 5% CO_2_. 

The tests were carried out in 24-well microplates using 4 × 10^4^ cells, cultured in 500 µL of DMEM + 10% FBS medium, and incubated at 37 °C with 5% CO_2_ for 24 h. After this period, the supernatant was discarded, and the cells were exposed to the treatments.

### 4.11. Treatment via Transwell

The treatments were carried out according to the groups: 500 µL of 3% HA; 500 µL of 1% KET; 500 µL of 3% HA + 1% KET; 500 µL of 3% HA + 0.500% KET; 500 µL of 3% HA + 0.250% KET; 500 µL of 3% HA + 0.125% KET; and 500 µL of DMEM + 10% FBS as a control. After 24 h of exposition, the treatments were discarded, and the wells were washed three times with PBS.

### 4.12. Cell Viability by Resazurin Essay

Metabolic activity was checked using resazurin at 440 µM. For this, 50 µL of the resazurin suspension was added to each well of the microplate, followed by the addition of 450 µL of DMEM + 10% FBS. Incubation in the dark was carried out for 16 h, after which the plate was read in a spectrophotometer at a wavelength of 570 nm. The optical densities (OD) obtained were converted into a percentage of cell viability using the following formula:

% Metabolic Activity = (OD Treated Group × 100)/Average OD Control Group

### 4.13. Genotoxicity Assay by Micronucleus

Mouse macrophages at a concentration of 3 × 10^5^ cells/mL were cultured in 96-well microplates with 1 mL of DMEM supplemented with 10% SFB for 24 h at 37 °C in a 5% CO_2_ atmosphere. The cells were exposed to the experimental groups under the same conditions. The negative control group received only the culture medium, while the positive control group received EMS at a concentration of 5 mM; both treatments were applied for 24 h. 

After the treatments, the cells were washed with PBS three times and incubated with cytochalasin B at a concentration of 6 μg/mL for 24 h at 37 °C in a 5% CO_2_ atmosphere. After the incubation, the cells were fixed in 100% methanol for 20 min, followed by staining with DAPI. The dye was removed after 5 min of contact with the cells, followed by three washes with PBS. Micronuclei were analyzed under a fluorescence microscope at 40X magnification with a total of 2000 cells evaluated per well.

### 4.14. Statistical Analysis

The data obtained was initially analyzed for normality using D’Agostino, Shapiro-Wilk, and Kolmogorov-Smirnov tests. Those that showed normality were then analyzed by ANOVA complemented by Turkey. Those that did not show normality were evaluated by Kruskall Wallys, complemented by Dunn’s test. All analyses utilized *p* value of 0.0001.

## Data Availability

The raw data supporting the conclusions of this article will be made available by the authors upon request.
